# Advances in Intranasal CNS Targeting: Integrating Formulations, Devices, Computational Fluid Dynamics, and 3D Printing

**DOI:** 10.3390/pharmaceutics18070902

**Published:** 2026-07-22

**Authors:** Lena Shaghlil, Yousef Al-Ebini, Mahmoud J. Al Shawabkeh, Fatmawati Adam, Kuldeep K. Saxena, Anas Alshishani, Wan Sharuzi Wan Harun

**Affiliations:** 1 Faculty of Mechanical and Automotive Engineering Technology, Universiti Malaysia Pahang Al-Sultan Abdullah, Pekan 26600, Pahang, Malaysia; linashaghlil93@gmail.com; 2 Center for Research in Advanced Fluid & Processes, Universiti Malaysia Pahang Al-Sultan Abdullah, Kuantan 26300, Pahang, Malaysia; fatmawati@umpsa.edu.my; 3 Department of Cosmetic Science, Faculty of Allied Medical Sciences, Al-Ahliyya Amman University, Amman 19328, Jordan; 4 Department of Oral Surgery and Diagnostic Sciences, Applied Science Private University, Amman 11937, Jordan; mahmoudalshawabkeh360@gmail.com; 5 Faculty of Chemical and Process Engineering Technology, Universiti Malaysia Pahang Al-Sultan Abdullah, Kuantan 26300, Pahang, Malaysia; 6 Department of Mechanical Engineering, Bennett University, Greater Noida 201310, India; saxena0081@gmail.com; 7 Research and Development Department, PharmaPrimes Lab, Amman 11190, Jordan; anasshishani@gmail.com; 8 Pharmaceutical Research Center, Zarqa University, Zarqa 13110, Jordan; 9 Faculty of Artificial Intelligence and Cybersecurity, Universiti Teknikal Malaysia Melaka, Durian Tunggal 76100, Melaka, Malaysia; 10 Department of Mechanical Engineering, Universitas Negeri Jakarta, Jl. Rawamangun Muka, Jakarta 13220, Indonesia

**Keywords:** nose-to-brain, nasal spray, nanoparticle carriers, mucoadhesive systems, smart polymers, 3D nasal casts, nasal devices

## Abstract

Nose-to-brain (N2B) delivery is a practical, non-invasive strategy for CNS targeting that can increase brain exposure while limiting systemic exposure. This review integrates three milestones in N2B delivery, formulations, devices, and quantitative evaluation strategies, to define design rules for effective olfactory/trigeminal deposition and enhance translational relevance. Formulations emphasize mucoadhesive systems, nanoparticle carriers (polymeric, lipid-based, and hybrid), nano-emulsions, and stimuli-responsive “smart” gels that prolong nasal residence. Regarding device advancements, the review covers conventional nasal sprays optimized for plume geometry and droplet size. Furthermore, it examines breath-actuated metered sprays, which promote soft palate closure to route aerosols to superior regions, and vibrating mesh nebulizers capable of low-velocity mists for improved upper cavity deposition. Quantitative evaluation is discussed, including 3D-printed, anatomy-accurate nasal casts, high-speed spray diagnostics, and computational fluid dynamics (CFD). This review further links formulation and device parameters to regional deposition. Available clinical and animal data illustrate the feasibility of these approaches, safety considerations, and user-technique dependencies, while highlighting the need for standardized, anatomy-aware testing protocols. Together, these developments suggest that co-designed formulation device platforms, validated by cast/CFD metrics and supported by clinical imaging or pharmacokinetic data, can support N2B product development toward consistent, patient-relevant outcomes.

## 1. Introduction

Neurological disorders represent one of the leading causes of disability and mortality, including conditions such as brain tumors, brain stroke, Alzheimer’s disease, and Parkinson’s disease [1]. Neurons are highly differentiated cells with unique structures and functions. As they possess limited regenerative capacity, CNS tissue is difficult to repair once damaged, creating a significant therapeutic challenge [2]. This delicate nature and uniqueness give the central nervous system (CNS), comprising the brain and spinal cord, the need for extra protection to limit the delivery of molecules into the CNS [3]. Delivering medicines to the CNS at a high concentration of the drug in the exact targeted area is more efficient using invasive administration methods such as intracerebral injection, intraventricular administration, and intrathecal injections [4,5]. Invasive administration methods require highly specialized professionals to avoid serious drawbacks such as hemorrhage, infection, and tissue damage, in addition to patient discomfort and limited feasibility for sustained treatments [6,7,8]. Alternatively, non-invasive drug delivery systems, such as transdermal, intranasal, pulmonary and oral delivery systems face many challenges such as systemic side effects, drug–food interactions, drug–drug interactions, low blood concentration due to systemic distribution, metabolic degradation, protein binding, and the most significant barrier, the blood–brain barrier (BBB) [3,9].

Nasal delivery provides an effective non-invasive route for therapeutic molecules to the CNS by the direct trigeminal and olfactory pathway. Although nasal delivery can avoid many limitations such as BBB and decreases systemic exposure it faces several limitations [10,11]. This review critically discusses key considerations in achieving effective nose-to-brain (N2B) drug delivery, with emphasis on recent advancements in intranasal administration strategies for targeted CNS therapies. The most promising formulation techniques, including mucoadhesive polymers, stimuli-responsive “smart” polymers, and nanoparticulate carriers, are highlighted for their potential to be combined strategically to enhance direct drug transport from the nasal cavity to the brain [12].

While previous reviews addressed formulations [13], device strategies [14], or CFD/cast evaluations separately [15], this review integrates evaluation strategies and emerging technologies to assess and optimize the deposition of combined formulation device approaches. Special focus is given to the role of digital and additive manufacturing (3D/4D printing, CFD) in advancing intranasal delivery toward clinical translation. Clinical insights are also discussed to highlight the practical implications and therapeutic promise of intranasal drug delivery systems for treating neurological disorders [16].

This review compiles recent data (2023–2025 studies) on 3D nasal casts/CFD data across multi-disciplinary fields (Pharmaceutical and engineering disciplines), creating a comprehensive roadmap for N2B developers by cataloging formulation device evaluation reported in the literature.

## 2. Methodology

To support this narrative review with a transparent evidence base, we searched articles from PubMed, Scopus, and Web of Science for the time records from 2000 to 2025. The keywords used were a combination of intranasal/nose-to-brain terms with formulation (mucoadhesive, nanoparticle, in-situ gel), device (breath-actuated, vibrating mesh), and evaluation (3D nasal cast, CFD, spray plume). We included peer-reviewed articles and reviews that reported formulation properties, device performance, deposition mapping, brain PK/PD, or nasal safety relevant to N2B using English language only. We excluded purely theoretical work without experimental validation.

## 3. Blood Brain Barrier

**Figure 1:**
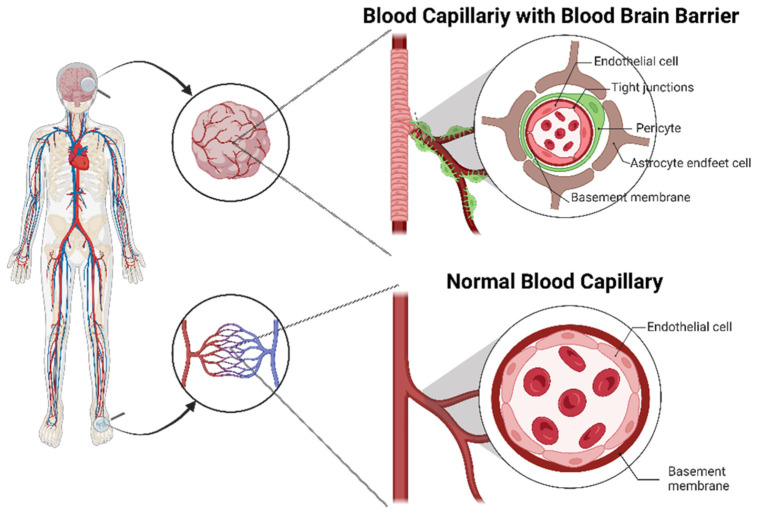
Schematic illustration of the structural differences between systemic blood capillaries and brain capillaries forming the blood–brain barrier (BBB).

Diseases related to CNS including Parkinson’s disease, brain tumors, meningitis, Alzheimer’s disease, and multiple sclerosis face significant challenges in delivering the drug to CNS neurons due to many limitations, but the main limitation that all conventional drug delivery systems (CDDS) such as oral, systemic, and transdermal must overcome is overcoming the BBB [17]. BBB is a highly specialized physiological boundary situated at the interface between the systemic circulation and the CNS, encompassing brain capillary endothelial cells and supported by a network of pericytes, astrocytic end-feet, and the basal lamina [18]. The BBB has special anatomical characteristics to guard the brain from foreign molecules, which restricts the delivery of most molecules including the drug, sometimes it is called the “protective shield” [19,20]. The BBB safeguards the CNS from potential damage by covering the blood capillaries surrounding the brain with an extra layer of cells as illustrated in Figure 1. Anatomically, the BBB is localized around the microvasculature of the brain, significantly restricting paracellular transport by tightly sealed intercellular junctions, known as tight junctions, formed by endothelial cells. Tight junctions primarily comprise proteins such as junctional adhesion molecules, occludins, and claudins, which provide selective permeability and high structural integrity to the endothelial lining [19]. Additionally, endothelial cells have minimal vesicular transport activity, lack fenestrations, and display significantly reduced pinocytic vesicle formation compared to peripheral endothelial cells, collectively reinforcing barrier selectivity [21].

Physiologically, the BBB functions as a critical homeostatic regulator of the CNS, precisely controlling the biochemical balance required for optimal neuronal function. It facilitates selective transport, allowing low molecular weight, lipophilic molecules like oxygen, carbon dioxide, and some lipid-soluble pharmaceuticals to diffuse across the barrier.

Specialized transportation systems, such as ion channels, glucose transporters, and amino acid transporters, are embedded in endothelial cell membranes to allow essential hydrophilic nutrients, such as glucose, ions, and amino acids, to pass across this barrier [22].

The BBB is armed with active efflux transporters like breast cancer resistance protein, P-glycoprotein, and multidrug resistance-associated proteins, which further limit drug permeability by actively extruding neurotoxic substances and xenobiotics from the endothelial cells into systemic circulation actively. These mechanisms support a robust physiological defense system that is essential for safeguarding CNS neurons from fluctuations in plasma concentrations, neurotoxins, and pathogens [23].

The robustness of the BBB is maintained primarily through the dynamic collaboration between endothelial cells, astrocytes, and pericytes. Astrocytes, with their end-feet structures, interact closely with endothelial cells, regulate BBB permeability, and support metabolic functions of endothelial cells by releasing signaling molecules [23]. Pericytes, embedded within the endothelial basement membrane cooperate in regulating endothelial proliferation, providing structural support, and modulating BBB permeability through signaling pathways involving angiopoietins and platelet-derived growth factors [24,25].

The BBB significantly obstructs the effective transport and distribution of many therapeutically active molecules, particularly macromolecules such as peptides, proteins, antibodies, and gene therapeutics. This selectivity represents a major challenge for CNS drug development, often demanding higher systemic dosages of therapies to achieve effective CNS concentrations, thereby risking significant systemic adverse effects and toxicity [26]. The subtherapeutic concentration of drugs in the CNS after the use of CDDS can be due to other factors such as tight junctions, high distribution volumes, metabolism, protein binding, variability in BBB permeability across individuals, and disease states, further complicating CNS drug delivery, predictability, and consistency [8,27].

To overcome or transiently pass the BBB, invasive approaches including intracerebral administration, direct intraparenchymal or intracerebroventricular infusion, intrathecal brain injection, and transient BBB disruption via focused ultrasound or osmotic techniques offer significant benefits in terms of local drug concentration but carry associated risks, such as neuronal damage, infection, hemorrhage, dose maldistribution, and the requirement of specialized personnel [28]. On the other hand, non-invasive delivery approaches, particularly intranasal administration, have emerged as promising alternatives by leveraging anatomical pathways like the olfactory and trigeminal nerves, bypassing the BBB and enabling direct CNS delivery. Intranasal administration facilitates rapid drug uptake and distribution within targeted brain regions, minimizing systemic exposure and potentially reducing adverse side effects compared with systemic routes [7].

## 4. Nasal Physiology

The nose extends from the nostrils to the choanae until reaching the pharynx. Intranasal drug delivery (INDD) has emerged as a preferred route for brain targeting because it offers a unique direct pathway that avoids the BBB [29]. To understand the uniqueness of the INDD, it is necessary to clarify the anatomy and physiology of the nasal cavity (NC). The nasal cavity, situated in the midface, lies inferior to the frontal sinus, superior to the oral cavity, and medial to the maxillary sinuses. As the most cephalic part of the respiratory system [30], it consists of two air-filled spaces separated by the nasal septum [30]. The upper third of the external nose contains paired nasal bones extending caudally from the frontal bone. The angle between the nasal and frontal bones is the nasofrontal angle. Each nasal cavity is further divided by three turbinates (superior, middle, and inferior), also known as conchae. These turbinates, projecting from the lateral walls, are rich in glands and blood supply [31]. The turbinates increase the surface area of the nasal cavity, thereby facilitating the warming and humidification of inspired air [32]. The total surface area is about 150 cm^2^, The most important anatomical feature of the NC is the relatively high surface area, in comparison to its size, which comprises three functional regions: olfactory, respiratory, and vestibule [12].

The vestibular region, anteriorly located, features squamous epithelium, hairs, and mucus, protecting the respiratory system from mechanical irritation and large particulate contaminants. The vestibule is lined with keratinized stratified squamous epithelium containing sebaceous glands, sweat glands, and coarse hairs [33,34]. The respiratory region, the largest, is lined with pseudostratified ciliated columnar epithelium and functions to warm and humidify the air. The respiratory region has a highly vascularized mucosa that promotes immediate drug absorption and systemic bioavailability [35,36]. The olfactory region, at the roof of the nasal cavity, is specialized for olfaction and provides a direct connection to the CNS via the trigeminal and olfactory nerves, thereby bypassing systemic circulation and the BBB [7]. Since it is the narrowest point, with cross-sectional area (3 cm^2^), the nasal valve, which is made up of the septum and the caudal end of the upper lateral cartilage, primarily produces turbulence. This, together with the acute turn of inspired air, adds to the high resistance to airflow [37].

The nasal mucosa contains ciliated cells, goblet cells, basal cells, and sensory neurons. Ciliated cells propel mucus, while goblet cells produce mucus to trap particles. Basal cells differentiate into ciliated or goblet cells; sensory neurons in the olfactory region detect odors. The nasal epithelium plays a vital role in drug absorption and mucosal immunity [31]. Because the nasal vasculature is permeable and dense, it facilitates the absorption of drugs throughout the body. Submucosal glands secrete mucus, maintaining a moist environment for mucociliary clearance. Mucus composition and rheology mainly affect drug residence time [38,39].

Mucociliary clearance is a crucial defense mechanism, removing inhaled particles and pathogens as cilia propel mucus towards the nasopharynx, where it is swallowed or expectorated. Mucociliary clearance and mucus permeation are the main limitations to nasal drug permeation; mucus consists of 95% water, 2% mucin proteins, some salts, and other molecules [40]. The nasal mucus is organized into two layers: gel viscous layer “mucus blanket” with 2–4 µm thickness, and light fluid layer “sol layer” with 3–5 µm thickness; the mucus moves by hook-shaped cilia known as “effective stroke motion”. This motion occurs when the gel layer moves along the fluid layer by 5–10 µm-long cilia beating at ~1000 strokes/min, as a result the nasal mucus transit speed from the interior part to the posterior parts of the NC in approximately 5 mm.min^−1^. Mucociliary clearance rates affect intranasal formulation efficacy as rapid clearance restricts residence time to approximately 15–20 min [41,42].

Despite the lack of routine clinical use of intranasal delivery for neurological disorders, several encouraging clinical developments highlight the translational potential of this route. Intranasal perillyl alcohol (NEO100) has advanced to Phase I/II clinical trials for recurrent malignant glioma, demonstrating tumor regression or stabilization with a well-tolerated safety profile [43,44]. More recently, intranasal foralumab, a fully human anti-CD3 monoclonal antibody, entered Phase II clinical investigation for neuroinflammation in Alzheimer’s disease following FDA expanded-access clearance in 2024 [45]. Beyond neurological disease, rapid systemic absorption via the intranasal route has proven clinically advantageous for acute conditions, with several FDA-approved products now in routine use for seizures, migraine, and opioid overdose [46]. While these approvals reflect systemic rather than direct N2B mechanisms, they validate intranasal administration as a clinically viable and rapidly effective delivery route.

## 5. Nose-to-Brain Limitations

The N2B pathway remained relatively unrecognized until the 1990s, when growing public and scientific interest in brain research prompted exploration of efficient treatment strategies for age-related neurodegenerative diseases [47]. It was then established that intranasal administration can facilitate the transport of therapeutic agents directly and indirectly to the brain via several distinct pathways. These pathways include the olfactory, trigeminal, and vascular routes, each with unique mechanisms, efficiency, and limitations.

**The vascular pathway** represents an indirect route for N2B delivery relying on the high vascularized nature of the nasal mucosa, where the drug reaches systemic circulation and can subsequently enter the CNS across the BBB [13]. The dynamics of systemic absorption depend on factors such as drug lipophilicity, molecular weight, and nasal blood flow. Although this pathway benefits from a large surface area and enables systemic drug distribution, its limitation lies in the BBB, which restricts the entry of many therapeutic agents. The vascular route is less targeted than direct neural pathways, leading to comparatively lower CNS drug concentrations [13,48].

**Figure 2:**
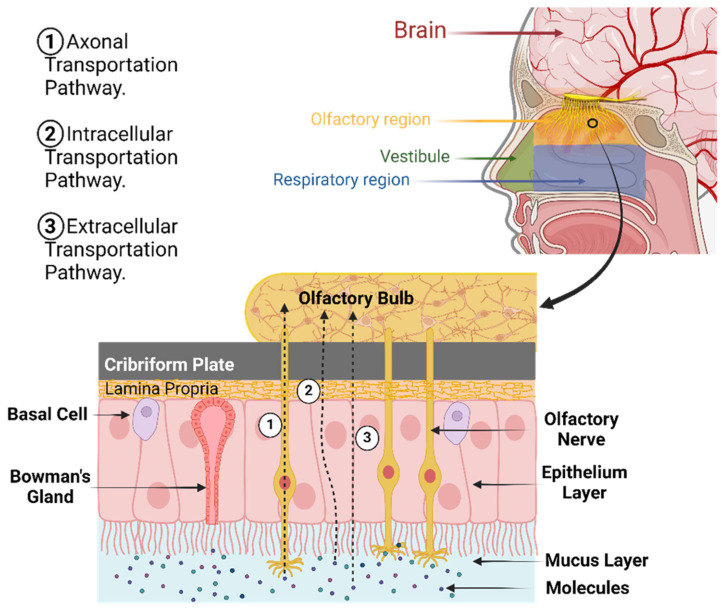
Schematic illustrating the main transportation pathways for N2B molecule delivery through the olfactory region.

**The olfactory pathway,** which is known as the shortcut to the brain, provides a direct connection to the CNS [49]. The olfactory area, located in the superior region of the nasal cavity is full of specialized olfactory neurons embedded within the nasal epithelium extending to reach the lamina propria layer and the cribriform plate until they finally reach the olfactory bulb, granting access to various brain regions, as shown in Figure 2 [3]. Drug transport through this region occurs via three main mechanisms: axonal, extracellular, and intracellular pathways (Figure 2) [42,50].

The **axonal transport route** is the slowest and occurs through the olfactory neuron along the axons, taking several hours or, in some cases, days for the molecules to finally reach the olfactory bulb. While this pathway offers direct access to the CNS, its efficiency is limited by factors such as enzymatic degradation and mucociliary clearance [12].

Intracellular and extracellular transport routes are considered a fast transportation pathway for molecules, and they are major transport mechanisms governed by diffusion. Both pathways are comparatively faster [51]. Extracellular transport occurs along perineural spaces surrounding the olfactory nerves, while intracellular transport involves uptake through epithelial cells, permeation across the mucosa, and eventual arrival at the olfactory bulb (Figure 2) [36]. Despite its direct access, the olfactory region is limited by its small surface area, anatomical location high in the nasal cavity, and rapid mucus clearance, all restricting N2B effectiveness [52,53].

The trigeminal pathway presents a direct route for N2B delivery, utilizing the extensive network of trigeminal nerve endings distributed throughout the nasal cavity, mainly in the respiratory region. The trigeminal nerve starts from the brainstem and divides into three main divisions: the maxillary, ophthalmic, and mandibular nerves. The trigeminal nerve can facilitate drug transport to the brainstem. Drug transport through the trigeminal nerve is currently being studied, although it most likely involves a combination of axonal transport and diffusion along nerve sheaths. Although the trigeminal pathway may have a greater surface area for drug absorption than the olfactory pathway, it also has a higher probability of taking a more indirect route (systemic absorption) to the brain [54].

The trigeminal pathways can cooperate in vaccination and treatment disorders with fewer side effects [13,55]. Another special feature of the trigeminal nerve is that it can be targeted using facial microneedles, as demonstrated in the delivery of rivastigmine to the brain using microneedle patches, which was found using male Wistar rats [56]. The therapeutic potential of the trigeminal nerve pathway in CNS delivery is significant, particularly for conditions affecting the cranial nerves and brainstem.

Despite the promising anatomical rationale underlying each of these pathways, it is critical to acknowledge that quantitative evidence for direct N2B transport efficiency remains highly variable and, in many cases, limited. Pharmacokinetic studies in rodents report direct transport percentages (DTP%) ranging from less than 45% for compounds such as antipyrine to greater than 95% for ranitidine and certain glycine receptor antagonists [57,58]. Crucially, some studies on other drugs, including insulin, oxytocin, progesterone, melatonin, and diazepam, have demonstrated no measurable pharmacokinetic advantage via intranasal over intravenous administration in primate and rodent models, indicating that N2B transport is highly drug-specific and governed by physicochemical properties such as molecular weight, lipophilicity, and charge [5]. However, for appropriately selected compounds, the evidence for direct N2B superiority is compelling; intranasal IGF-I produced CNS concentrations more than 100-fold higher than matched intravenous dosing, with biological activity confirmed at target sites [59]. Glycine receptor antagonists demonstrated DTP values of 99.99% and 96.71%, with the drug physically confirmed throughout the olfactory nerve apparatus within one minute of dosing [58]. Donepezil formulations further achieved a direct delivery ratio of 80.32% with brain concentrations exceeding twice those of systemic routes [60]. These findings collectively suggest that N2B transport efficiency is highly drug-dependent, being greatest for hydrophilic, BBB-impermeable compounds, and protein therapeutics. Furthermore, the olfactory epithelium in humans occupies a substantially smaller surface area than in rodents, limiting direct extrapolation of preclinical findings to clinical outcomes. Quantitative human pharmacokinetic data for the N2B route remains critically lacking in the literature, making it premature to assert reliable therapeutic efficacy for most neurological indications at this stage. The present review therefore frames the described formulation, device, and evaluation strategies as tools to maximize the potential of a pathway whose clinical translation still requires rigorous, quantitative human validation.

## 6. Formulation Strategies

Various technologies and strategies have been evaluated to improve N2B targeting. The two primary categories of recently employed technologies, namely device-related and formulation-related technologies, are both focused on improving the longevity of the active therapeutic molecules at the target location and increasing drug penetration, which in turn increases the drug’s availability in the brain.

Formulation development is essential to achieve the required therapeutic effect, by controlling the drug release and physiochemical properties [61]. Formulation-related strategies rely mainly on the use of mucoadhesive polymers [62], nanoparticle technology [63], smart polymer technology, and permeation enhancers [64]. Formulation strategies used for N2B formulations are aimed at enhancing the efficiency of delivering the drugs to the brain, such as rapid clearance for the mucus layer covering the NC, protect the active pharmaceutical ingredient from enzymes in the mucosal layer, and address the critical position of the olfactory region [65]. Formulation strategies highlighted in this part are the most commonly used and efficient strategies in N2B studies [66]. The use of mucoadhesive polymers can notably increase the attachment time of the formula and resist the effect of mucus clearance, whereas the use of nanoparticles enhances the stability and the permeability of the active drug, and the use of smart polymers support the adaptation of the formula to the NC conditions, which affects the drug release profile and residence time [67].

### 6.1. Mucoadhesive Polymers

Mucoadhesive and thermoresponsive systems have garnered considerable interest among advanced formulation strategies for N2B drug delivery due to their ability to enhance nasal residence time, reduce mucociliary clearance, and promote sustained and targeted delivery of therapeutics to the central nervous system (CNS) [68,69]. These systems rely on polymers that interact physically or chemically with the mucosal surface, thereby improving the formulation’s retention in the nasal cavity and increasing the opportunity for drug absorption, which increases bioavailability [70]. Mucoadhesive polymers utilize both natural and synthetic polymers to create strong bonds with the nasal mucosa. The polymer adheres to the nasal mucosa by ionic bonding, hydrogen bonding, hydrophobic interactions, electrostatic interactions, or physical entanglement with the mucus layer. This adherence resists mucociliary clearance, thereby enhancing the efficiency of drug transport [71]. These polymers adhere to the mucosal surface and slow down drug elimination through mucociliary clearance, increasing drug bioavailability in the brain and prolonging contact time, especially for medications with short half-lives or those that need rapid and efficient CNS penetration [72].

Natural mucoadhesive polymers have proven to be stable, safe, economical, and abundant, have a high rate of hydration, and provide a significant effect in resisting mucociliary clearance [73]. Chitosan, which is one of the most widely used polymers, is a naturally occurring, mucoadhesive polymer that is positively charged and derived from chitin. It is nontoxic, biodegradable, and acts as a permeation enhancer in nasal formulations; it can temporarily open tight junctions in epithelial tissue [74]. Also, it has a short gelation time with primer adhesion properties [75]. Chitosan emulsion showed a significant increase in the residence time and compared with a drug solution for ovalbumin delivery [55]. It was also found to increase the adhesion and stability of the formulation after addition to a freeze-dried powder used for nasal brain targeting. Other commonly used polymers include Carbopol, hydroxypropyl methylcellulose (HPMC), sodium alginate, Carboxymethyl cellulose (CMC), polyacrylic acid, and hydroxyethyl cellulose (HEC). These agents are effective at enhancing the viscosity of nasal patch formulations and improving their retention and deposition in the nasal cavity [76,77]. Synthetic mucoadhesive polymers such as Pluronic F-127 and Soluplus^®^ proved their efficacy at forming a firm mucoadhesive system in nasal and ocular drug delivery [78,79]. Other mucoadhesive polymers proved the prolonged residence time after using them such as the use of hydroxypropyl-β-cyclodextrin [80,81]. Mucoadhesive formulations provided shorter Tmax and increased the brain’s drug amount compared with the blood plasma [82]. Even the use of different grades of HPMC had significant effect on the release, adhesion, and permeation properties of nasal insert formula [83]. Lyophilized inserts made of HPMC and mannitol [84] and chitosan gellan gum polyelectrolyte were used in nasal insert formulations and proved to enhance adhesion and bioavailability [85]. The effectiveness of mucoadhesive formulation technology was proven using several animal models, such as monkeys, as it was shown that N2B delivery was significantly improved after evaluation of in vivo cynomolgus monkey and 3D printed magnetic resonance imaging (MRI) nasal cast for monkey and human noses. This was proved for a powder formulation delivered using pressurized air newly designed device, whereas the liquid formulation was delivered using a commercial MAD Nasal™ Device (Teleflex Medical, Wayne, PA, USA) [86].

### 6.2. Nanoparticles Carrier

The application of nanotechnology in nasal drug delivery has significantly advanced strategies aimed at targeting the central nervous system (CNS) through the intranasal route. Nanoparticle formulation technology is widely used due to its ability to provide very small particles that offer chemical stability for drugs, penetrate small capillaries, enhance mucosal permeation of water-insoluble drugs, encapsulation of high molecular weight molecules, prolong residence time, and control drug release [10,87]. Nanoparticles used in N2B delivery can be classified broadly into two systems (inorganic and organic). Inorganic nanoparticles, such as gold nanoparticles, iron oxide particles, mesoporous silica nanoparticles, and carbon-based materials, provide unique advantages in terms of imaging compatibility and structural stability [49,88]. Gold nanoparticles allow surface modification and theranostic applications but raise concerns about accumulation and long-term safety. Iron oxide nanoparticles can be magnetically guided to enhance targeting, while mesoporous silica nanoparticles offer tunable pore structures for controlled drug release. Despite these benefits, inorganic systems often lack biodegradability and may induce oxidative stress or inflammation [49,89]. Nanoparticles loaded with levodopa showed significant improvement in Parkinson’s symptoms and higher drug concentration compared with oral drug administration [90]. The most commonly used nanoparticle types for N2B are lipid-based nanoparticles, polymeric nanoparticles, and nano-emulsions [91]. Dry powder nanoparticles can also be formulated using various techniques [92]. Technologies such as supercritical fluid extraction, milling, and spray drying have different capabilities for controlling particle size, surface properties, and morphology, all of which critically affect deposition and absorption efficiency [92,93].

Nanoparticle technology prolongs the attachment/residence time of the drug by increasing the adhesion force using electrostatic adsorption. Electrostatic adsorption results from the negatively charged mucins in the mucus and positively charged polymers that form nanoparticles [94]. Chitosan nanoparticles, whose mucoadhesive and biodegradable properties are detailed in Section 6.1, are the most commonly used nanoparticle system for N2B delivery, as their cationic character enables electrostatic adhesion to the nasal mucosa [95]. It has been reported that chitosan-coated nanoparticle, compared to intravenous injection of carmustine, showed a 2-fold enhancement of drug permeation, and a 15-fold enhancement for AUC_0-t_ in Albino Wistar rats [96]. Another work proved that the drug concentration in the systemic circulation using chitosan nanoparticles was lower than free drug administration due to the increased diffusion of sitagliptin to the brain [97]. Chitosan nanoparticles were shown to improve brain targeting by 2.6 times in Wistar rats and to increase penetration by more than 70% in just 24 h using goat nasal tissue [98].

**Organic nanoparticles** include solid lipid nanoparticles (SLNs) and nanostructured lipid carriers (NLCs), polymeric nanoparticles, micelles, liposomes, and dendrimers [99]. Lipid-based nanoparticles are preferred due to their biocompatibility, biodegradability, ability to encapsulate both hydrophilic and lipophilic drugs, capacity for surface modification to add specific targeting proteins, and protective characteristics that prevent drug degradation in the nasal environment [100,101]. SLNs are composed of solid lipids and exhibit good stability, although their limited drug loading capacity and potential for polymorphic transitions remain concerns [102]. NLCs, which incorporate a mixture of liquid and solid lipids, offer improved loading efficiency and better long-term stability [101,103].

Solid lipid nanoparticles (SLNs) and nanostructured lipid carriers (NLCs) mainly consist of lipophilic molecules, which enhance active and passive transport throughout the nasal epithelium. Active transport includes permeation through receptor-mediated, adsorptive, or transporter-mediated transcytosis. Passive transport includes transmembrane diffusion or paracellular transport due to the nano size of the nano-emulsion [104]. Loading Piribedil in solid lipid nanoparticle thermoresponsive methyl cellulose gel showed a 4-fold increase in brain availability and 2.3-fold decrease in the plasma (C_max_) in comparison with a plain intranasal suspension [105]. Liposomes are spherical vesicles composed of one or more phospholipid bilayers enclosing an aqueous core, enabling encapsulation of hydrophilic drugs within the core and lipophilic drugs within the bilayer [106]. Some research studies have demonstrated that liposome-loaded drugs have a high potential for safe and effective treatment of Alzheimer’s disease, as shown by cell differentiation tests and cytotoxicity studies. These studies indicate that liposome formulations not only deliver the drug efficiently but also minimize toxicity to healthy cells, supporting their potential as a therapeutic strategy for Alzheimer’s treatment [107]. Additionally, it was observed that PEG-modified liposomes significantly enhanced brain and spinal cord permeation, for particles with a size of 100 nm, as demonstrated by fluorescence imaging. This effect was confirmed after the nasal administration of liposomes labeled with fluorescence, highlighting the potential of these reconfigured nanoparticles for targeted CNS drug delivery [108].

**Polymeric nanoparticles** constructed mainly from biodegradable polymers such as poly (lactic-co-glycolic acid) (PLGA), polylactic acid (PLA), or chitosan provide superior control over drug release profiles and particle size [49]. Chitosan-based systems demonstrate mucoadhesive properties and have been shown to enhance paracellular transport by transiently opening epithelial tight junctions [102]. However, the batch variability of natural polymers and the potential immunogenicity of synthetic alternatives must be considered [55]. Particles, generally less than 200 nm in size, are useful for brain delivery. Despite their high surface functionality and drug-loading ability, dendrimers face limitations owing to their complex synthesis and cytotoxicity risks [65].

The primary components of polymeric nanoparticles are poly (lactic-co-glycolic acid) (PLGA), polylactic acid (PLA), and polyglycolic acid (PGA). PLA is a low-water-soluble polymer with weak mechanical strength, while PGA is a high-water-soluble polymer with low stability. Meanwhile, PLGA is a bulk-eroding polymer that is more hydrophobic, which causes slower degradation rates because of its methyl side groups. This hydrophobicity contributes to its sustained release properties, making it a suitable material for controlled drug delivery applications [109]. In a comparative study, it was observed that PLGA nanoparticles demonstrated smaller particle sizes, and increased drug loading compared to solid lipid nanoparticles (SLNs). Additionally, PLGA nanoparticles exhibited a better sustained-release profile. Both PLGA nanoparticles and SLNs showed superior results in in vitro studies and are expected to provide enhanced brain distribution in in vivo applications [110].

**Nano-emulsions** consist of two immiscible liquids, water (W) and oil (O) with the addition of a surfactant and/or cosurfactant; they can have two forms of nano-emulsion depending on the surfactant used and the quantity of oil and water, O-in-W (O/W) and W-in-O (W/O). The formulation of nano-emulsions is usually performed using two techniques with low and high energy. The phase inversion temperature method is used as a low-energy technique, whereas high-energy techniques include the microfluidic technique, ultrasonication, and homogenization using high pressure [111,112]. Nano-emulsions have been shown to efficiently target brain cancer following nasal administration, significantly enhancing systemic circulation permeability and absorption for the drug. This approach bypasses the metabolism in the liver, therefore reducing drug toxicity and improving the overall therapeutic drug efficacy [113,114]. The most commonly recommended type of emulsions for nasal formulations is the O/W emulsion, as it has the advantages of enzymatic protection and reduces pH-mediated degradation. It was shown that O/W nano-emulsion gives suitable sustained release properties with accepted viscosity and a high stability and safety profile [114,115]. Another study found that a medication incorporated into a nano-emulsion exhibited significantly higher nasal ex vivo permeability and in vitro drug release, with a direct transport percentage of 76.17% [116].

### 6.3. Smart Polymers

Smart polymers utilize stimuli-responsive polymers, which represent a class of materials that undergo reversible physical or chemical changes in response to specific environmental/external triggers. These triggers, which include, ionic strength, temperature, light, pH, specific molecules, magnetic, and electric fields, induce changes in the physical properties of the polymer driving conformational changes [117]. This change can be swelling, shrinking, or a change in viscosity. The ability of smart polymers to adapt dynamically to physiological conditions makes them highly valuable in advanced drug delivery systems, especially in targeting the central nervous system (CNS) via the intranasal route [118]. In N2B delivery, smart polymers enhance formulation performance by improving drug stability, residence time, and bioavailability while enabling controlled or targeted release [119]. Smart polymers can be broadly categorized based on the stimuli they respond to. The most common types include thermoresponsive polymers, pH-sensitive polymers, and dual-sensitive systems that respond to both temperature and pH [120]. Thermoresponsive polymers such as poloxamer 407 (Pluronic F127) are liquid at room temperature but form a gel upon contact with the nasal mucosa due to body heat (~32–34 °C). pH-sensitive polymers like polyacrylic acid and cellulose acetate phthalate gel or dissolve at specific pH levels, allowing for site-specific release. Dual-sensitive polymers combine the benefits of both systems to enhance formulation robustness and responsiveness to complex nasal conditions [121].

The selection of smart polymers depends on multiple factors, including the physicochemical properties of the drug, the desired release profile, compatibility with nasal mucosa, and formulation scalability. Thermoresponsive hydrogels like poloxamer-based systems are often favored for their simplicity and excellent in situ gelling behavior. However, their drug retention may be limited for highly hydrophilic molecules. pH-sensitive polymers provide better control in inflamed or pathological mucosa but may exhibit slower gelation kinetics. Smart polymers’ main advantages are enhanced patient compliance, prolonged drug contact with the absorption site, and reduced dosing frequency. Disadvantages may include formulation complexity, potential mucosal irritation, and cost-related scalability issues [122]. Hydrogels and thermogels are particularly effective in increasing nasal drug retention. Hydrogels form a crosslinked three-dimensional network that holds water and drug molecules, enabling sustained release. Thermogels, which undergo gelation upon warming, can adapt to the nasal environment and resist mucociliary clearance, thereby maximizing contact with olfactory and trigeminal pathways. This prolongs the window for drug absorption into the CNS and minimizes systemic dispersion [65].

Gellan gum is an ionic-responsive smart gelling polymer that can transition from solution to gel phase after nasal administration [123]. In situ gel efficiency for rivastigmine using chitosan and carbopol 934 has exhibited increase in efficiency by 7-fold compared to a normal intranasal rivastigmine solution [124]. Smart polymer technology has been merged with nanoparticle technology for advanced brain targeting when administered nasally. When comparing oral administration with thermosensitive nanoparticles, it has been proven that thermosensitive nanoparticles show higher concentrations of the drug in brain tissue (flurbiprofen) [125]. Thermosensitive polymers combined with nano-emulsion to create a nanoemulgel using the low-energy emulsification method resulted in a highly stable formulation. This formulation provides constant drug release and significantly increases drug permeability across RPMI-2650 cells [126]. An ion-sensitive nano-emulsion formulated for N2B drug delivery demonstrated a 1.6-fold inhibition of tumor growth and a 1.2-fold improvement in the survival rate of nasally treated rats in the treatment of glioblastoma tumors [127].

Smart polymers enable 4D-printed constructs that respond to stimuli; 4D printing refers to the behavior of the printed object, not the material itself. Four-dimensional printing is an enhancement of 3D printing because it incorporates smart materials that can alter shape or functionality over time in response to external stimuli [128]. The 4D term refers to the fourth dimension, which can be temperature, time, humidity, or pH, which determines the dynamic behavior of the stimuli-responsive printed material, such as hydrogels, and shape-memory polymers [129]. Four-dimensional printing technology allows the creation of flexible structures that can adapt to the environment [128]. Four-dimensional printing is an interesting future potential for N2B delivery. It is expected that 4D printing technology can be used to create personalized nasal formula or specific drug carriers that adapt to the NC’s shape and environment or respond to specific physiological triggers. The advantages of structural transformation and the release of drugs at specific targeted sites within the NC could significantly improve the precision and effectiveness of N2B delivery, particularly for conditions that require controlled release and targeted delivery [128]. One of the key benefits of 4D printing in this context is its potential to improve patient compliance and comfort by allowing for minimally invasive and self-adjusting devices that optimize drug delivery based on the patient’s unique anatomical and physiological characteristics [130]. The advantages and disadvantages of each formulation technology are summarized in Table 1.

The recent use of previously mentioned formulation technologies in in vivo studies is summarized in Table 2, as it shows the use of technologies and their advancement in studies using different animal models.

Additionally, all previously mentioned formulations were administered as nasal sprays or nasal drops. Nasal drops, sprays, and powder formulations face deposition challenges, which are influenced by factors such as surface charge, droplet size, plume geometry, and spray pattern [131,132]. Herein, the interest in developing nasal devices increased. The recent rise of nasal devices relies on improving the efficiency and reproducibility of olfactory targeting and depositing of drug administration [133]. Other technologies used for nasal delivery are nasal device optimization and administration methods for various nasal formulation types that have been utilized for N2B.

**Table 1:** Advantages and disadvantages of different formulation types used for N2B drug delivery.

Formulation Type	Advantages	Disadvantages
Mucoadhesive Polymers	Prolonged nasal residence time: Mucoadhesive properties increase contact time with nasal mucosa, enhancing drug absorption [134]. Enhanced permeability: Improve drug permeation across nasal epithelium through tight junction modulation [134]. Improved mucoadhesion: Strong adhesion to nasal mucosa prevents rapid clearance [135] Biocompatibility: Generally recognized as safe with minimal toxicity concerns [135].	Mucociliary clearance: Despite adhesion, still subject to natural clearance mechanisms [134]. Potential nasal irritation: High polymer concentrations may cause local irritation [136]. Variable mucus layer: Effectiveness depends on mucus composition and thickness, which varies among individuals [136].
Lipid-Based Nanoparticles (SLN/NLC)	Enhanced lipophilic drug encapsulation: Excellent for poorly water-soluble drugs [137]. High biocompatibility: Composed of physiological lipids with minimal toxicity [135]. Controlled and sustained release: Lipid matrix enables prolonged drug release [135]. Protection from enzymatic degradation: Lipid shell protects encapsulated drugs [138]. Scale-up advantage: Cheap and easy to scale-up for large-scale production [137].	Limited drug loading capacity: Especially for hydrophilic drugs [135]. Physical instability: Prone to particle aggregation and gelation during storage [135]. Polymorphic transitions: Lipid crystallization can lead to drug expulsion [135]. High surfactant concentration: May cause nasal irritation and toxicity [139].
Polymeric Nanoparticles	Sustained drug release: Controlled release profiles over extended periods [135]. Drug protection: Shield drugs from premature biodegradation and enzymatic attack [137] Surface modification potential: Easy functionalization with targeting ligands and cell-penetrating peptides [135]. Versatility: Accommodate both hydrophilic and hydrophobic drugs [135]. Biodegradability: Polymers like PLGA and chitosan are biocompatible and biodegradable [135]. Enhanced permeability and mucoadhesion: Improve drug transport across biological barriers [135].	Burst release: Initial rapid drug release can reduce therapeutic efficacy [135]. Potential toxicity: Some synthetic polymers may cause inflammatory responses [139] Scale-up challenges: Difficulty maintaining reproducibility in large-scale production [136,140]. Regulatory hurdles: Complex characterization requirements for approval [140].
Nano-emulsions	Enhanced drug solubility: Improve solubilization of lipophilic drugs [141] Improved nasal permeation: Small droplet size facilitates absorption across nasal epithelium [141]. Thermodynamic stability: More stable than conventional emulsions [135].	Physical instability: Susceptible to Ostwald ripening and phase separation [135]. Limited drug loading: Restricted capacity for high drug concentrations [139]. High surfactant concentration: May cause nasal irritation [139].
Liposomes	Excellent biocompatibility: Composed of natural phospholipids similar to cell membranes [135]. Versatile drug encapsulation: Can encapsulate both hydrophilic (aqueous core) and lipophilic (bilayer) drugs [137]. Membrane fusion capability: Facilitate direct drug delivery into cells [137]. Surface modification: Easy conjugation with targeting moieties and mucoadhesive polymers [135].	Rapid clearance: Quick elimination from nasal cavity due to mucociliary clearance [135]. Low drug loading: Limited encapsulation efficiency, especially for hydrophobic drugs [135]. Potential immunogenicity: Some polymers may trigger immune responses [135].
Hybrid/Combined Systems	Synergistic effects: Combine advantages of multiple formulation types [136]. Improved targeting: Multifunctional design enables better brain specificity [136]. Multifunctionality: Simultaneous drug protection, mucoadhesion, and controlled release [136]. Higher brain-to-serum ratios: Superior brain accumulation compared to single systems [142].	Formulation complexity: Intricate design and optimization requirements [139] Characterization challenges: Difficult to fully characterize multi-component systems. Regulatory uncertainty: Unclear approval pathways for novel hybrid systems [139]. Limited long-term stability data: Insufficient information on storage stability [139].

**Table 2:** Recent in vivo studies (2023–2025) using advanced formulation technologies for N2B delivery and key outcomes.

Formulation State	Technologies Used	Therapeutic Target	Drug Used	Technology Application Advancement	Animal Model Used	Year	Ref
Liquid	In-situ gel + Nanoparticles (starch nanoparticles)	Bipolar disorder	Lithium	Controlled release for up to 6 h. lower drug levels in serum. High cytocompatibility.	Adult male Sprague-Dawley rats	2025	[143]
Liquid	Nanoparticles (Phytosomes)	Alzheimer disease	Ginseng + Rivastigmine hydrogen tartrate	Significant synergistic effect for combination treatment. Nanoparticles compared to IV and oral, shows highest Cmax, AUC0-∞, and a Tmax very close to IV.	Sprague-Dawley rats	2025	[144]
Liquid	Nanoparticles (Bilosomes)	Anti-migraine	Rizatriptan	Formulation shows 2.94 times prioritized bioavailability. sustained release profile (96.41% over 24 h)	Albino Wistar rats	2025	[145]
Powder	Mucoadhesive polymers + microparticle(spray dried)	Tuberculosis	Rifampicin + Isoniazid	Formula shows high safety profile regarding cytotoxicity and cell viability. Significantly reduced the mycobacterial load in the brain (~0.78 Log10 CFU reduction).	BALB/c mice	2025	[146]
Liquid	Mucoadhesive polymers + Nanoparticle (Chitosan nanoparticles)	Schizophrenia	Lurasidone hydrochloride	The use of chitosan nanoparticles enhances the sustained release profile for the drug. Conjugation of nanoparticles to n transferrin increase drug accumulation in the brain tissue.	Wistar rats	2025	[147]
Liquid	Mucoadhesive polymers	Depression and panic disorder	Alprazolam	High safety profile. Shorter onset of action, and a longer duration compared to oral.	C57BL mice	2025	[148]
Liquid	In-situ gel + Nanoparticle (NLC) + Mucoadhesive polymers	Alzheimer disease	Nifedipine	Advanced neuroprotective efficacy in terms of behavioral, biochemical, and histopathological examination. Prolonged release, up to ~24 h. Superior stability.	Wistar rats	2024	[142]
Liquid	In-situ gel	Alzheimer disease	Flurbiprofen	Formulation shows three times brain bioavailability (Cmax = 490.3 ng/mL) over oral (Cmax = 145.1 ng/mL). Shorter Tmax in the brain for nasal formulation over oral Ansaid^®^. In-situ gel proven its safety, and efficacy to replacement oral formulations.	Sprague-Dawley rats	2024	[125]
Powder	Mucoadhesive polymers	Alzheimer disease	Insulin	Micro-sized dry powder influences the drug distribution in the brain.	Wistar rats	2024	[149]
Liquid	Nanoparticle (Micelles)	Multiple sclerosis	Ibudilast	Micelles formulation resulted in a higher drug concentration in the brain over oral and free drug solution. Micelles increase in myelin fiber density in the corpus callosum in the brain	C57BL/6 mice	2023	[150]
Suspension	Mucoadhesive polymers	Anti-epilepsy	Carbamazepine	Using amorphous solid dispersion increases drug transfer to brain for in vivo tests. Amorphous solid dispersion enhances dissolution profile for in vitro test.	Male Wistar rats	2023	[151]
Suspension	Mucoadhesive polymers	Nausea and vomiting	Domperidone	The enhancement of mucoadhesive polymers for N2B was proved using 3D printed monkey and human nose and using in vivo monkey experiment.	Cynomolgus monkey	2023	[86]
Powder	Nanoparticle (nano spray drying) + Mucoadhesive polymers	Alzheimer disease	Galantamine	The nanosized particles enhance the distribution in the olfactory rejoin. The mucoadhesive polymers increases the residence time, but it was more effective when combined with permeation enhancer.	Swiss mice	2023	[152]
Liquid	Nanoparticle (Micelles)	Brain tumor	Methotrexate	Micelles show high chemical stability for the drug. Micelles increase the penetration of the drug to the brain	New Zealand White rabbits	2023	[153]

Smart polymers are mainly used to increase the contact time between the formulation and the nasal mucosa to assure maximum time for drug permeation; this contact may cause irritation and/or toxicity, depending on the nature of the polymer and the drug composition, in comparison with the conventional nasal solution [154]. Local irritation, ciliotoxicity, tissue destruction, and epithelial or subepithelial toxicity are common concerns with sustained release intranasal formulations [123,155].

## 7. Device Strategies

Nasal devices used in nasal delivery systems can be utilized for three key purposes: local treatment within the internal nasal parts, fast-acting systemic administration, and direct N2B delivery. Generally, the targeted nasal pathways are affected mainly by the deposition of the drug inside the NC, which can be controlled by adjusting some device parameters [156]. Therefore, several device strategies have been developed to optimize the deposition and distribution of the drug within the NC. Sprays, breath-actuated metered sprays, and vibrating mesh nebulizers are the primary devices used effectively in medicine delivery to the brain, and they will be discussed below.

### 7.1. Nasal Sprays

Nasal sprays are widely used, non-invasive devices for nasal administration, offering local, systemic, and brain delivery. They are effective for treating local conditions by delivering medication directly to the nasal mucosa, reducing symptoms with minimal side effects [157]. For systemic administration, nasal sprays provide rapid drug absorption by bypassing the gastrointestinal tract and liver metabolism, offering super short T_max_. In brain delivery, nasal sprays can target the brain via the trigeminal or olfactory nerves, making them promising for neurological treatments [158]. The sprayed formulation can be a solution, suspension, or powder. Nasal spray proved its efficiency in treating local symptoms and reducing side effects such as nasal allergic rhinitis sprays [159]. Targeting properties of the nasal spray rely on many factors such as spray angle, spray velocity, droplet size, and the spray geometry, which affect the deposition and droplet size distribution of the drug inside the NC [160]. Nasal sprays demonstrate rapid onset of action for systemic drug delivery and reduce drug–drug interactions compared with oral solutions; therefore, they enhance the pharmacodynamic and pharmacokinetic profiles of the drug, as stated for midazolam [161]. A study evaluating the distribution of residual drug concentration across various organs including heart, liver, kidney, lung, and nasal mucosa for a muscarinic receptor blocker revealed that the highest drug concentration persisted in the nasal mucosa for over 24 h post-administration of the nasal spray, as demonstrated in dogs and rats [162].

The main characteristics responsible for spray aerosol deposition in the NC can be divided into two main factors: formulation and device-related factors. The formulation-related factors are viscosity of the formulation and droplet/powder size distribution [163]. Evaluation of the drug distribution after nasal spray administration in the brain for new formulation of rivastigmine with the Exelon^®^ oral capsule proved that the viscosity had a critical effect on the droplet size and, therefore, the deposition of the formula, which directly affects the brain targeting efficacy, as it was found that the optimum viscosity value for achieving olfactory deposition is ~77 mPa⋅s [164]. A recent study used extended interferometric particle imaging to evaluate droplet size and droplet location, and a high relation was observed between the spray pattern and the refractive index, which have the highest contribution to the nasal spray efficiency [165]. Another study found that different particle sizes result in different deposition sites inside the NC and different absorption percents. The most efficient droplet size for targeting the anterior part of the NC is 30–100 µm, while 60–180 µm gives the best deposition in the upper part of the NC, which aligns perfectly with the FDA recommendations for nasal sprays [166].

### 7.2. Breath-Actuated Metered Spray

The breath-actuated metered spray (BAMS) synchronizes the delivery of drugs with controlled exhalation through the mouth, which enhances the deposition of drug molecules in the NC, especially in olfactory region deposition and reduces dose loss [167]. On the other hand, traditional nasal sprays deliver a fixed dose regardless of the patient’s breathing pattern [168]. The mechanism of this device relies on a pressure-sensitive valve that only opens during mouth exhalation, allowing drug deposition in the NC only, which optimizes both the site and timing of drug absorption. Blowing air from the mouth (oral exhalation) naturally elevates the soft palate upward to achieve good closure with the nasopharynx, which locks the formulation inside the NC, allowing the release of the formulation into the airstream and giving the formulation a chance to be deposited throughout the NC [169].

The primary advantage of BAMS over traditional nasal sprays is increased drug olfactory deposition. This is achieved by delivering the drug during optimal air dynamic conditions, which minimizes drug loss due to exhalation and swallowing [170]. BAMS appears to increase patient compliance, as it provides a user-friendly and highly intuitive administration method in comparison to the traditional spraying method [170]. Despite the complexity of the device, the need for precise synchronization for the breath pattern of the patient and the potential drawback of drug loss.

**Figure 3:**
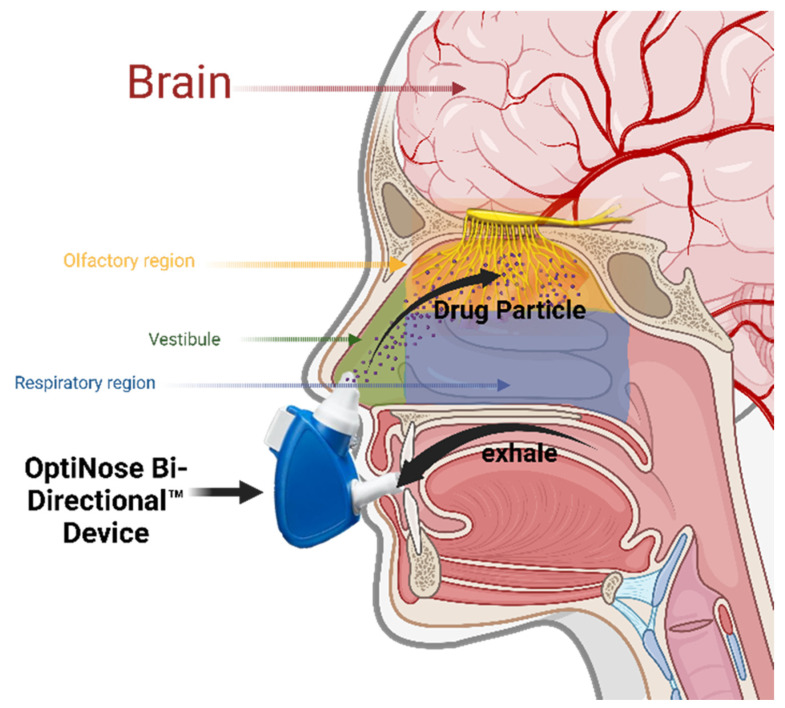
Illustration of drug particle deposition of drug powder all over the nasal cavity (NC) while using breath-actuated metered spray (BAMS) represented with the OptiNose device.

The most commonly known examples of the BAMS in the market are the OptiNose Bi-Directional™ device (Optinose US Inc., Yardley, PA, USA) for powder formulations [171] and XHANCE^®^ for liquid formulations [172]; both are defined as unique devices with enhanced mechanisms that employ the patient’s exhaled breath to deliver the drug to the top/inner most part of the NC [171]. The OptiNose Bi-Directional™ device is used to administer 11 mg of sumatriptan powder, which is mainly used for migraine headache patients. Sumatriptan is used to fill a chamber in a one-use nosepiece as a standard respiratory capsule; when the depressing button is activated, the drug capsule is crushed. Exhaling air into the device rotates and vibrates powder in conjunction with the exhaled air, releasing the drug into the NC for delivering drugs [170]. The mechanism of this device relies on the pressure difference between the oral and nasal cavities; as the positive pressure created by the oral exhalation in the oropharynx, this difference in pressure allows good particle deposition in the upper NC as illustrated in Figure 3 [170].

Clinical studies by Djupesland et al. (2012) evaluated and compared the deposition pattern of traditional nasal spray and a breath-actuated powder device in healthy volunteers, and the results showed that BAMS reduced lung inhalation, significantly enhanced deposition in the upper NC, and provided more friendly administration for the patients [173]. Another study by Djupesland et al. (2004) evaluated the deposition of sumatriptan in the lungs after the use of BAMS and a regular nasal spray, and the results demonstrated the increased safety of BAMS and decreased the risks of lung disease and airway obstruction [174]. Although clinical studies have proven the priority of BAMS over regular nasal sprays, its performance over intravenous (IV) treatment has not yet been evaluated.

BAMS proves its superior effect over conventional nasal sprays, as it provides a high drug concentration in the olfactory region [175]. On the other hand, this administration method still suffers from mucociliary clearance, patient’s ability to use the device properly, and enzymatic degradation, although it still offers a fast onset of action and an accelerated absorption rate in comparison to oral drug administration [169].

### 7.3. Vibrating Mesh Nebulizer

The vibrating mesh nebulizer (VMN) is a device used for N2B delivery, by generating a very fine mist using an aperture plate to carry drug molecules to the olfactory region [176]. Traditional jet nebulizers, which deliver drugs as very fine aerosols to the lungs, generate aerosols using compressed air. In contrast, VMN produces a mist using a vibrating piezoelectric crystal at high frequencies, which propels the drug solution through a mesh composed of multiple laser-drilled holes. Finally, this process results in a fine aerosol low velocity capable of reaching the olfactory region [163].

**Figure 4:**
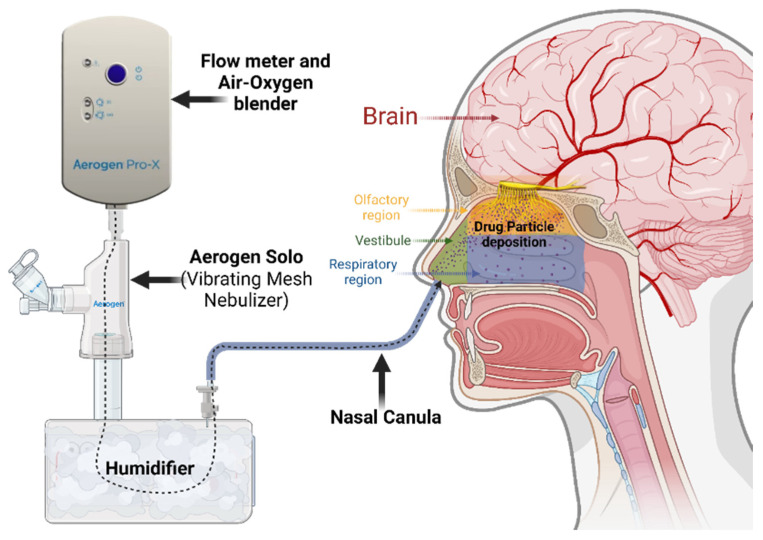
Illustration for the vibrating mesh nebulizer (VMN) device components.

The main advantage of VMNs is their ability to generate a large number of respirable particles suitable for olfactory deposition. These respirable particles have a cutoff diameter of 5 μm [177]. Due to their small diameter, these very fine mists can reach the deepest regions of the nasal cavity, including the olfactory region. The small mist size enhances both bioavailability and absorption via the nose-to-brain route. This process decreases dose deposition in the lungs and improves efficacy [178]. Despite this device’s advantages, its complexity and the potential clogging of the mesh or aperture plate are considered major drawbacks. Additionally, the high likelihood of drug loss in the device chamber and the increased chance of deposition in the lungs are important limitations. There are many examples of VMN devices; however, the most well-known device is the Aerogen^®^ Solo (Aerogen, Galway, Ireland) [179]. The Aerogen^®^ Solo is a portable, single patient-use device that generates aerosol using a vibrating mesh and delivers the mist via a facemask or nasal cannula, as illustrated in Figure 4.

A clinical study investigated and compared the regional deposition of aerosol generated by a VMN versus a jet nebulizer in the NC and lungs of non-smoking, healthy male volunteers using a gamma camera. The results showed that the VMN delivered a threefold increase in dose to the NC compared to the traditional jet nebulizer, whereas the jet nebulizer resulted in a 27% increase in lung deposition [180]. Another clinical study applied on seven non-smoking, healthy male volunteers aged 21–36 years, as confirmed by scintigraphic imaging, demonstrated that 73 ± 10% of an aerosol loaded with 99mTc-DTPA was deposited in the upper airways, when a sound system (100 Hz) was coupled with a nasal nebulizer during 10 min of nebulization. These findings indicate that a nasal sonic jet nebulizer can be successfully used for drug delivery to the NC and sinuses [181].

Although VMNs have demonstrated advantages for nasal cavity deposition compared to conventional nebulizers, direct evidence of N2B drug transport was not measured in these studies, and human quantitative N2B pharmacokinetic data remain critically lacking. Additionally, there is a high risk of dose loss from both the container and the device, and further development is required to reduce lung deposition. Furthermore, the high cost of the device cannot be ignored.

## 8. Evaluation Strategies

Evaluation strategies for N2B are fundamental to ensuring the safety and efficacy of delivering drug molecules to the target region. These strategies primarily include in vivo and in vitro approaches. Often, a combination of these methods is employed, utilizing advanced technologies to align in vitro findings with in vivo results. This integration enables more accurate and reliable evaluations [182].

The technologies used in these strategies focus on critical parameters such as deposition patterns, spray dynamics, and particle size. These evaluations are essential for optimizing drug delivery systems and ensuring effective targeting of the desired regions in NC.

### 8.1. Deposition Evaluation Technology

The evaluation of particle deposition within the NC is one of the most critical aspects of N2B research, as nasal deposition significantly influences the pharmacokinetics of the drug [182]. The complexity and uniqueness of the NC anatomy pose challenges for accurately positioning drug molecules. To address these challenges, several advanced technologies have been developed to enhance the accuracy and efficiency of deposition in some studies, as no standard methodology was recorded to evaluate the drug deposition in nasal delivery studies.

Among these, 3D printing technology plays a pivotal role in evaluating nasal deposition. This technology is employed to create customized and commercially available nasal casts that provide highly accurate anatomical representations of NC [17]. These casts are instrumental in understanding deposition patterns and optimizing drug delivery systems. Additionally, 3D simulations of the NC are utilized to model deposition behaviors under various conditions, further refining delivery strategies and improving therapeutic outcomes [183].

### 8.2. Customized 3D Nasal Casting

Three-dimensional printing, or additive manufacturing, is the process of building objects in 3D from digital models [184]. This technology provides substantial control in the development and assembly of structures, making it well suited for medicinal and biological usage [184]. There are mainly three types of 3D printing methods that are related to drug delivery: Stereolithography (SLA), Fused Deposition Modeling (FDM), and Inkjet Printing [185]. SLA uses laser or UV light to harden photopolymer resins [186]. FDM uses thermoplastic materials, which are formed through extrusion of thin filaments of thermoplastic material through a heated nozzle to successively lay down layers of material to create the object [187], whereas Inkjet Printing involves spraying droplets of material or cells on layers [188]. Three-dimensional printing can be useful in different approaches such as formulation, organ casting, and personalized devices. The main benefits of 3D printing are the high flexibility of form customization to the patient’s and drug’s needs, the development of complicated shapes, contributing to better dispersion and absorption, and fast iteration of designs for testing and dosage formulation [189]. This customization capability is very useful, especially for N2B, due to the potential ability in targeting the olfactory region, which is responsible for delivering drugs to the brain without passing through the blood–brain barrier.

The use of 3D printing in formulation strategies is highly used in oral formulations, as it provides an opportunity to create dosage forms that release the active pharmaceutical ingredient in a controlled manner [190], by printing a 3D shell over the tablet [191], or creating an air-pocket for a gastroretentive tablet to increase the residence time in the stomach and ensure drug release for more than 12 h [192]. In the field of nasal drug delivery, 3D printing is extensively utilized for organ casting techniques. This technology has been applied in the in vitro evaluation of N2B delivery, significantly advancing the development of NC casts [193]. Nasal molds are specifically developed based on imaging data from real patients, allowing for the reproduction of nasal structures with high precision. These models serve as realistic tools for assessing drug delivery systems [167,194]. Several 3D printing techniques have been used to build precise duplicates of human airway geometries, which are crucial for investigating drug deposition and optimizing particle size and distribution [195].

Three-dimensional printing enables the comparison of different nasal cavities (NCs) across various population groups. For instance, a study comparing the deposition of nasal aerosols between adult and pediatric NCs using fluticasone furoate revealed that pediatric models provided valuable insights into nasal spray performance evaluation [196]. Another study utilized 3D-printed nasal casts to target olfactory drug delivery, demonstrating that anatomical differences significantly influence the deposition efficiency of powdered drugs [197,198]. These findings highlight the necessity of controlled drug delivery systems.

Additionally, research using nasal casts has investigated spray patterns and the deposition of liquid formulations and nasal sprays, influenced by varying spray device designs and formulation viscosities. These studies have shown how differences in these factors affect drug dispersion and absorption, contributing to the development of more effective nasal sprays. This, in turn, enhances drug targeting to the olfactory region of the NC and improves bioavailability [199,200]. For example, one study examined three commercial nasal sprays on healthy and rhinitis humans, proving the effectiveness of 3D printing in the assessment of formulation deposition and spray distribution [201]. A huge study has evaluated the anatomical dimensions of 40 healthy pediatric (2–11 years old) nasal cavities to investigate the most influential parameters for targeting properties, which were evaluated using two different nasal spray products. The model was connected to a breathing simulator to have more accurate and realistic evaluation for plume geometry, droplet size distribution, and spray pattern [202,203].

Moreover, 3D-printed nasal casts are invaluable for understanding how specific anatomical characteristics, such as obstacles or deviations, impact drug delivery. This facilitates the development of optimized equipment and formulations tailored to individual patients [204]. These patient-specific nasal casts add significant value to in vitro studies by considering anatomical differences for precise deposition mapping [205]. Overall, 3D printing has proven to be an indispensable tool for advancing nasal drug delivery research and improving therapeutic outcomes.

Three-dimensional printing can be used for maxillofacial prostheses; casting of the nose can be performed using molds or direct printing [206]. In a case report, it was recorded that 3D printing for a 27-year-old woman, who was provided with a nasal prosthesis because of a traffic accident, required significantly require less time, enhanced reproducibility and acceptability in the production of the nasal prosthesis, as the described 3D printing workflow proved, when compared to the same stages followed by the conventional procedure [207]. Another case study proved the superiority, for a 22-year old female with postsurgical nasal defect and using the mold technique [208].

Three-dimensional bioprinting is a completely novel approach for generating cells, tissues, and organs, combining biological elements with superior print equipment to create detailed constructions that have similarities to tissue. It has strong application prospects in the fields of regenerative medication, drug development, and precise treatment [209].

### 8.3. Commercial 3D Nasal Casts

The availability of commercial 3D NCs is important for education and research evaluations. Commercial 3D nasal casting reduces the time, effort, and cost, in some cases, to achieve accurate representative human nasal passages. Both GTSimulators^TM^ (Davie, FL, USA) and SOMSO-PLAST ^®^ (Coburg, Germany) offer human nose and sinus models that are mostly used as educational models [210,211]. GTSimulators^TM^ provide a detailed cross-sectional view for anatomical representations of the NC and sinus that are widely used for clinical training and educational purposes to understand the intricate features of the physiological and pathological nasal passages [210]. Similarly, SOMSO-PLAST ^®^ offers high-quality detailed craftsmanship and is used for medical education; they offer many models such as nose alone, nose and NC model, cavities of the nose, mouth, and throat with larynx, and cavities of the nose, mouth, and throat with larynx. SOMSO-PLAST ^®^ offers the nose of a cow model besides the different human models [211].

Koken transparent NC model, produced by Koken Co. (Tokyo, Japan), Ltd. [212], Aeronose^®^ Nasal CAST [213], and Alberta Idealized Nasal Inlet Model [214] are commercial nasal casts that can be used in Copley scientific research. The Koken model or cavity model LM-005 is constructed from transparent silicone, which allows a clear view for the internal details of the NC. This transparency is highly beneficial for educational purposes and for deposition pattern evaluation, which is used in nasal formulation evaluation [215]. The Koken model is separated into two unidentical halves aligned with the nasal septum, which allow clear comprehensive examination for nasal turbinate and other internal anatomical structures [216]. The Koken LM-005 model was used to assess the transmission efficiency of atomized water particles all over the NC; the evaluation was in an advanced experiment setup, as they add the bronchial tube model. The experiment successfully studied the behavior of viscous and non-viscous liquid in a condition that mimic medical nebulization; the relation between the viscosity and particle size directly affects the deposition and distribution of atomized particles [217].

The Aero nose^®^ Nasal cast was co-developed by Aptar Pharma (Crystal Lake, IL, USA) [213]. Aeronose is a 3D-printed model constructed for comprehensive presentation of the unique areas in the NC, such as the nasal valve, turbinate, and rhinopharynx. Aeronose is considered an advanced in vitro model designed to support the development of nasal spray applications, specially N2B-targeted formulations [213]. In a study, Aeronose was used to evaluate the device type and parameters on the deposition of valproic acid loaded in a rhodamine B-labeled nano-lipid, which indicated that the nanoparticle has advanced potential for protecting and delivering valproic acid effectively, and appropriate spray setup can significantly increase the fraction of dose deposition [101].

The Alberta Idealized Nasal Inlet Model is another commercial NC model available that was developed by the Aerosol Research Laboratory of Alberta [214]. Alberta Idealized can be expressed as a standardized geometry model for NC; it is highly used in nasal formulation evaluation studies, not only nasal formulations as sprays and aerosols, but also inhalers and formulations designed for lung targeting therapy by connecting the nasal cast to a next generation impactor as an extension [213]. The Alberta nasal cast has a flexible design, which allows it to separate in a vertical part for more accurate evaluation of each part. The most critical areas shown accurately by the Alberta nasal cast are the outer cartilaginous part of the nose, nasal valve, ethmoidal bone, floor of NC, three turbinates, and rhinopharynx [214]. The Alberta nasal cast was successfully used in deposition evaluation of engineered nanoparticles containing transforming growth factor-β in to the nose to treat cerebrovascular diseases, where a scanning electron microscope (SEM) was used to evaluate the size distributed in each part of the NC [218]. Alberta was used to evaluate the powder formulations as in favipiravir assessment for N2B delivery, as it has proven that using spray freeze drying enhances the delivery of class II drugs by 1.57 fold to have in the olfactory region and reduces the drug loss to the lungs and throat, and adding isonicotinamide to the formulation increases the olfactory drug deposition by enhancing the particle size distribution and adhesive properties by forming a hybrid intermolecular network of hydrogen bonds, including amide-amide homosynthon and amide-pyridine heterosynthon [219]. A further study was constructed to evaluate the deposition of rivastigmine loaded in an in situ hydrogel nanosystem lipid. Using the Alberta nasal cast model revealed that the best percent deposition was 4% for both rivastigmine-loaded lipid-based nanoparticles and nano-emulsion, where the deposition for the in situ hydrogels shows a 2-fold increase (8%) in rivastigmine. All formulas showed the most deposition in the vestibule and turbinate regions [220]. Comparison of the commercially available nasal cast models, SOMSO-PLAST^®^, Alberta Idealized Nasal Inlet Model, Aeronose^®^ nasal cast, and Koken cavity model LM-005, is shown in Table 3.

Even though 3D printing can be costly and does not stimulate the exact NC conditions, especially the dynamic of air flow inside the NC. Three-dimensional printing requires accurate and precise printing material with high-performance printers. Therefore, some researchers have started using 3D simulation software.

### 8.4. Computational Fluid Dynamics Nasal Simulation

Three-dimensional simulation involves the use of special software to simulate the air flow and the dynamic movement of particles inside the NC using computational fluid dynamics (CFD) models [221]. CFD was used to study the particle deposition and movement through all the respiratory airways, especially the lung; then, it was used to evaluate the interindividual differences of the NC [221]. Employing high-flow auxiliary gas (HAG) methods reveals that airflow distribution alternated by the changes in congestion and decongestion surpass natural breathing deposition [222]. The simulation evaluation relies on critical variables, including particle size, nozzle insertion depth, and administration angle [223].

The use of CFD in recent studies reveals that better fluidity will be for smaller particle sizes. Small particle sizes can traverse through the curved nasal pathways to reach the olfactory area with higher efficiency [223]. Simulations reveal that adjusting the auxiliary airflow rates affects the deposition and dispersion competing forces and enhances small particle delivery while minimizing wastage to the lungs. In particular, increasing the auxiliary airflow from 5 to 30 L/min has been shown to increase the deposition of the olfactory rate by ~four-fold [224]. The use of 3D casting and 3D simulation is summarized in Table 4 [225]. Table 4 reveals consistent patterns in N2B deposition evaluation. Freeze-dried powders [219,226] enhance olfactory targeting, while conventional sprays are primarily deposited in vestibule/turbinate regions (8%) [220]. CFD confirms that small particles and airflow optimization improve targeting. Evidence remains inconsistent for patient-specific CT casts due to variable resin materials and a lack of information to link the material variability, affecting model realism.

A recent study proved a tight correlation among olfactory deposition, droplet size, viscosity, and plume geometry. The in vivo pharmacodynamic experiments and pharmacokinetics provide rigid proof for enhancing N2B delivery [144]. Therefore, the importance of spraying evaluation cannot be ignored, and the use of technology enhances brain targeting.

### 8.5. Spraying Evaluation

The efficiency of N2B delivery is heavily influenced by the parameters derived from both formulation and device, particularly for sprayed formulations (liquid and powder), which include spray angle, droplet size, and plume geometry. Adjusting any of these parameters has a direct effect on the drug’s reach to the olfactory area and, therefore, brain bioavailability [235]. Nasal spray devices are now developed to achieve precise control over these parameters to increase drug deposition in target areas.

Spraying device evaluation relies on device-related factors, which have a primer and crucial role for drug deposition in NC. The cone angle is the most critical factor, followed by characteristic droplet size, plume ovality, spraying uniformity and velocity constant [236]. The angle tip and the spraying position for nasal spray were examined for their critical role in drug deposition within the NC, and it was found that a straight spray tip will result in more deposition of particles in the upper nasal regions (olfactory area) [237]. In another study on the effect of auxiliary airflow velocity and spraying cone angle on particle deposition using computational fluid dynamics, a 40° cone angle was the most favorable for the low auxiliary airflow, but the spray cone angle did not show any effect for 20 L/min auxiliary airflow or more, whereas a 60° spray cone angle showed the best deposition regarding the auxiliary airflow [228]. A further study evaluated the spray cone angle for a unidose powder spray using VEO4K camera (Vision Research, Wayne, NJ, USA) with 1000 frames/s for formulated donepezil powder and proved that a larger particle size decreases the spraying cone angle, which enhances drug targeting in the NC [238]. Spraying characteristics of nasal human insulin solution were investigated using two commercial spray pumps. The study used a 3D printed nasal cast for the in vitro evaluation and MRI for human volunteers; it was proven that the ideal plume angle should be between 30° and 45° to obtain the best olfactory deposition [239]. As demonstrated by the evaluation of the nasal steroid spray using CFD models, droplet size is a critical factor that influences drug deposition and needs to be balanced. After examination, for droplet sizes from 5 to 1030 μm, it was found that particles with low momentum are more affected by airflow, which directly affects their trajectory and deposition location, which means that very small droplets may not be deposited efficiently, while those that are too large may not penetrate the nasal passages adequately [201].

In contrast to the substantial preclinical literature, quantitative human evidence for the N2B route remains sparse and inconsistent. Born et al. provided the only direct pharmacokinetic evidence, showing that intranasal insulin (40 IU) accumulated in the CSF from 10 min, peaked at 30 min, and remained elevated at 80 min, while plasma stayed stable [240]. In contrast, Lowe et al. gave intranasal insulin lispro at doses up to 160 IU in eight volunteers, yet CSF levels stayed below quantification at all doses despite being detectable in beagle dogs [241]. The largest trial to date further found that 40 IU daily for 12 months produced no cognitive or functional benefit over placebo [242]. These mixed outcomes partly reflect anatomical and device limitations. The human olfactory epithelium covers less than 10% of the nasal surface versus roughly 40–50% in rodents, and modeling predicts that nasal nanomaterials reach the mouse brain at two orders of magnitude more than the human brain [12]. Overall, while intranasal delivery is feasible and well tolerated in humans, robust quantitative confirmation of clinically meaningful N2B delivery is still lacking.

## 9. Conclusions and Future Perspective

In conclusion, N2B drug delivery is a promising approach for CNS and brain targeting. After accurate study and investigation of the uniqueness of NC anatomy and physiology, especially the olfactory area, pharmaceutical science and technologies can innovate remarkable formulation and devices that significantly improve drug transportation to the brain, which will highlight the potential for all neuronal disordered patients. The involvement of nasal devices is promising and holds potential for future impact on clinical practice, particularly for neurological disorders where clinical translation remains limited despite two decades of preclinical advancement, with ongoing clinical investigations such as intranasal foralumab for Alzheimer’s disease and intranasal perillyl alcohol for glioma representing encouraging early steps toward routine clinical application. Future studies are expected to focus more on 3D printing with biodegradable and biocompatible materials, which will advance the potential of 4D printing technologies. Additionally, collaboration between pharmacists, engineers, and clinicians can have great advantages to overcoming the drug delivery limitation and ensure effective translation from the lab to the clinic. Finally, nasal drug delivery is still full of gaps and interesting research opportunities.

## Figures and Tables

**Table 3:** Comparison of commercially available nasal cast models used in nose-to-brain drug delivery research and education: SOMSO-PLAST^®^ [210], Alberta Idealized Nasal Inlet Model [213], Aeronose^®^ nasal cast [212], and Koken cavity model LM-005 [211].

Model	Manufacturer	Material	Key Features	Application	Ref
SOMSO-PLAST^®^	SOMSO	---	Educational; multiple configurations	Medical education	[211]
Alberta Idealized	Alberta Aerosol Lab	---	Standardized geometry; separable; connects to NGI	Spray/aerosol/inhaler evaluation	[214]
Aeronose^®^	Aptar Pharma	3D-printed	Shows nasal valve, turbinate, rhino pharynx	Nasal spray/N2B development	[213]
Koken LM-005	Koken Co.	Transparent silicone	Splits at septum; transparent	Deposition evaluation	[212]

**Table 4:** Summary for recent (2023–2025) research papers that used 3D casting and 3D simulation for N2B delivery.

Evaluation Method	3D Printing Method	Source of the NC	Material Used for Printing	Drug Used	Formulation Type	Ref
3D Casting	FDM	Trapezoidal geometry computer-aided design NC	Polyvinyl chloride (PVC) flexible	Favipiravir	Freeze Dried Powders	[219]
	STL	CT scans for Chinese adults and Goats	Transparent resin and ordinary resin	-	Freeze Dried Powders	[226]
	STL	CT scans of 20 subjects	The posterior section: high clarity rigid plastic The anterior section: flexible rubbery material	Triamcinolone Acetonide	Suspension	[227]
	STL	CT scans of 20 subjects	Clear rigid plastic (Accura ClearVue)	Triamcinolone Acetonide	Suspension	[196]
	STL	CT scan of healthy young Asian	Transparent resin rigid and Tango as flexible material	Stain	Solution	[228]
	NA	CT scan of a 62-year healthy patient	All parts: transparent rigid Accura ClearVue except the anterior region: flexible plastic	Diazepam	In-Situ Hydrogel	[229]
	STL	CT scan obtained from hospital	Formlabs Clear^®^ resin	Caffeine	Solution	[200]
	NA	anonymized CT scan of healthy adult man	NA	Ginseng + Rivastigmine	Solution	[144]
**Evaluation method**	**Year**	**Source of the NC**	**Software used**	**Drug Used**	**Formulation Type**	**Ref**
3D Simulation	2025	Nine healthy individuals	ANSYS-FLUENT 2021	Mometasone Furoate	Gas	[230]
	2025	CT scan of a 7-year-old child	OsiriX Software	Steroids	Solution	[231]
	2025	CT scan for female subject	SpaceClaim 17.2	Oxycodone Hydrochloride	Powder	[232]
	2025	MRI image for 11 healthy subjects	ANSYS ICEM-CFD	NA	Solution	[233]
	2024	CT scans for children from 2 to 11 years	Velocity-controlled Vereo^®^: side-ctuator (Vereo SSx) + top-actuator (Vereo NSx)	Fluticasone Furoate	Solution	[203]
	2023	MRI healthy male (28 years old)	ANSYS ICEM-CFD	NA	Powder	[234]

## Data Availability

Data sharing is not applicable to this article, as no datasets were generated or analyzed during the study.
